# Global liver gene expression differences in Nelore steers with divergent residual feed intake phenotypes

**DOI:** 10.1186/s12864-015-1464-x

**Published:** 2015-03-25

**Authors:** Polyana C Tizioto, Luiz L Coutinho, Jared E Decker, Robert D Schnabel, Kamila O Rosa, Priscila SN Oliveira, Marcela M Souza, Gerson B Mourão, Rymer R Tullio, Amália S Chaves, Dante PD Lanna, Adhemar Zerlotini-Neto, Mauricio A Mudadu, Jeremy F Taylor, Luciana CA Regitano

**Affiliations:** Embrapa Southeast Livestock, São Carlos, SP Brazil; Division of Animal Sciences, University of Missouri Columbia, Columbia, MO USA; Department of Animal Science, University of São Paulo/ESALQ, Piracicaba, São Paulo Brazil; Department of Animal Science, State University of Sao Paulo, Jaboticabal, SP Brazil; Department of Genetics and Evolution, Federal University of Sao Carlos, São Carlos, SP Brazil; Embrapa Agricultural Informatics, Campinas, SP Brazil

**Keywords:** *Bos indicus*, RFI, Feed efficiency, Transcriptomics

## Abstract

**Background:**

Efficiency of feed utilization is important for animal production because it can reduce greenhouse gas emissions and improve industry profitability. However, the genetic basis of feed utilization in livestock remains poorly understood. Recent developments in molecular genetics, such as platforms for genome-wide genotyping and sequencing, provide an opportunity to identify genes and pathways that influence production traits. It is known that transcriptional networks influence feed efficiency-related traits such as growth and energy balance. This study sought to identify differentially expressed genes in animals genetically divergent for Residual Feed Intake (RFI), using RNA sequencing methodology (RNA-seq) to obtain information from genome-wide expression profiles in the liver tissues of Nelore cattle.

**Results:**

Differential gene expression analysis between high Residual Feed Intake (HRFI, inefficient) and low Residual Feed Intake (LRFI, efficient) groups was performed to provide insights into the molecular mechanisms that underlie feed efficiency-related traits in beef cattle. A total of 112 annotated genes were identified as being differentially expressed between animals with divergent RFI phenotypes. These genes are involved in ion transport and metal ion binding; act as membrane or transmembrane proteins; and belong to gene clusters that are likely related to the transport and catalysis of molecules through the cell membrane and essential mechanisms of nutrient absorption. Genes with functions in cellular signaling, growth and proliferation, cell death and survival were also differentially expressed. Among the over-represented pathways were drug or xenobiotic metabolism, complement and coagulation cascades, NRF2-mediated oxidative stress, melatonin degradation and glutathione metabolism.

**Conclusions:**

Our data provide new insights and perspectives on the genetic basis of feed efficiency in cattle. Some previously identified mechanisms were supported and new pathways controlling feed efficiency in Nelore cattle were discovered. We potentially identified genes and pathways that play key roles in hepatic metabolic adaptations to oxidative stress such as those involved in antioxidant mechanisms. These results improve our understanding of the metabolic mechanisms underlying feed efficiency in beef cattle and will help develop strategies for selection towards the desired phenotype.

**Electronic supplementary material:**

The online version of this article (doi:10.1186/s12864-015-1464-x) contains supplementary material, which is available to authorized users.

## Background

Feed efficiency-related traits are increasingly being studied because of their importance to the overall profitability of animal production. Moreover, the selection of more efficient animals reduces the land required for feed production, methane emissions and nitrogen excretion resulting from the digestion/metabolic process [1-3]. Heritability estimates for feed efficiency-related traits are moderate in dairy and beef cattle [4-7], including the Nelore breed [8]; however, genetic variation for feed efficiency has not been widely exploited in animal breeding programs because the measurement of this trait is costly [1].

There are several indices that are commonly used to estimate the feed efficiency of growing cattle; one of them being residual feed intake (RFI) which is independent of body weight and weight gain. RFI is used to identify individuals that deviate from their expected level of feed intake given their size and growth rate over at least a 70 day feeding period [3]. Because RFI is not phenotypically dependent on the production traits that are used to estimate expected feed intake, it is possible to compare RFI among individuals that differ in their level of production. This independence has led some researchers to believe that RFI may reflect intrinsic variation in basic metabolic processes [9].

Developments in molecular genetics, specifically high-throughput sequencing methods, offer a unique opportunity to identify genes and pathways that are associated with complex traits and diseases [10]. Current DNA and RNA sequencing methodologies are becoming important tools for unravelling the mechanisms which underlie complex traits, facilitating a new understanding of the genetic regulation of phenotype and allowing for the identification of potential biomarkers for early or more accurate genetic prediction. Gene expression profiling can be applied to identify differentially expressed (DE) genes and isoforms involved in networks that control complex traits, thereby shedding some light on the molecular mechanisms responsible for variation in target traits.

Recent studies have identified putative quantitative trait loci (QTL) for feed efficiency on several chromosomes in Nelore populations [8,11]. However, these studies have largely identified discordant genomic regions, revealing a limitation of genome-wide association studies (GWAS) for identifying loci with significant effects within different subpopulations of the same breed [12]. In this research, two divergent groups of Nelore cattle were selected on their best linear unbiased predictions (BLUP) of additive genetic merit for RFI and classified as either high (HRFI) or low (LRFI). RNA sequencing was used to profile the gene expression of hepatic tissue of 20 sampled animals.

## Results

### Sequencing throughput, read mapping, and assembly

The RFI phenotypes for this Nelore population were previously used to perform a genome-wide association study (GWAS) and the summary statistics for the population were described [8]. Table 1 presents the BLUP estimates of additive genetic merit, phenotypes, sequencing throughput and mapping statistics for each sample used in this study.

**Table 1:** **Best Linear Unbiased Predictions (BLUP) of additive genetic merit for Residual Feed Intake (RFI), dry matter intake (DMI), average daily gain (ADG), sire, number of reads passing filter and concordant pair alignment rate for each animal within the Low (LRFI, efficient) or High (HRFI, inefficient) groups based on RFI BLUP estimates**

**Animal_ID**	**Phenotype**	**BLUP (Kg/day)**	**RFI (Kg/day)**	**DMI (Kg/day)**	**ADG (Kg/day)**	**Sire**	**Reads passing filter**	**Concordant pair alignment rate (%)**
NE003327	LRFI	−0.0914	−1.0493	7.75	1.7	NE003322	9,761,212	92.3
NE003343	LRFI	−0.0699	−0.5469	8.75	1.41	NE001360	12,689,051	92
NE003344	LRFI	−0.036	−0.5714	8.45	1.48	NE001388	8,324,143	91.8
NE003349	LRFI	−0.099	−1.2284	8.49	1.73	NE001383	9,476,944	92
NE003350	LRFI	−0.0862	−0.7682	8.57	1.75	NE001360	8,179,991	91.4
NE003363	LRFI	−0.0414	−0.6588	7.43	0.98	NE001382	11,090,049	92.5
NE003364	LRFI	−0.0341	−0.3803	8.38	1.39	NE001380	10,369,298	92
NE003377	LRFI	−0.0417	−0.1459	10.1	1.83	NE001391	10,209,752	91.9
NE003464	LRFI	−0.0679	−1.1983	8.4	1.78	NE003323	9,821,754	92
NE003473	LRFI	−0.0306	−0.2845	10.41	2.33	NE001359	9,570,163	91.9
Mean		−0.0598	−0.6832	8.47	1.715		9,949,236	91.98
NE003352	HRFI	0.0856	0.327	8.97	1.66	NE001707	10,736,571	91.7
NE003355	HRFI	0.0939	0.6588	10.07	1.54	NE001360	8,304,145	92.5
NE003368	HRFI	0.0876	0.4115	9.72	1.86	NE001390	11,240,045	92.6
NE003393	HRFI	0.048	0.2443	9.36	2.06	NE001383	12,166,612	92.4
NE003398	HRFI	0.0721	−0.1548	9.78	1.78	NE003322	8,778,347	92.5
NE003416	HRFI	0.1247	1.8084	10.53	1.39	NE001388	10,473,989	92
NE003431	HRFI	0.0875	0.4206	10.32	1.92	NE001394	9,370,303	92.1
NE003439	HRFI	0.0688	−0.2976	8.92	1.77	NE001391	10,588,391	92
NE003456	HRFI	0.0861	1.2807	9.09	1.53	NE001382	9,238,005	92.2
NE003498	HRFI	0.0924	0.5969	10.17	1.85	NE003323	10,059,686	92.2
Mean		0.0847	0.5296	9.75	1.775		10,095,609	92.6

After mapping reads with TopHat v2.0.6 [13,14], Cufflinks v2.0.2 [14,15] was used to assemble the transcriptome for each sample. The Cuffmerge utility was then run to create a unique file which contained a parsimonious set of transcripts for these data. The number of detected transcripts that represented potentially new isoforms was very large (~71.44% of the transcripts); nevertheless this was expected considering that almost all genes in mammals undergo alternative splicing [16]. We found a total of 16,962 annotated genes to be expressed in bovine liver; however, 5,707 rare or highly expressed (>1 million reads) genes were not tested in the analysis for differential expression. Lowly expressed genes cannot be statistically tested by the Cuffdiff 2 algorithm while the analysis of highly expressed genes leads to excessive machine memory demands [14,15].

To evaluate sequence quality, we assessed the distribution of transcript abundances for each expressed gene as a box-plot of the log of FPKM values (Additional file 1: Figure S1). Very similar median and quartile values for FPKM estimates were observed for the members of both RFI groups. We also evaluated the expression profiles of selected housekeeping genes Hypoxanthine Phosphoribosyltransferase 1 (*HPRT1*) and Tyrosine 3-Monooxygenase/Tryptophan 5-Monooxygenase Activation Protein, Zeta (*YWHAZ*) and found expression patterns for these genes to be similar within each of the treatments. Finally, a principal component analysis (PCA) of FPKM values for all genes indicated that there were sufficient numbers of DE genes to differentiate the RFI groups (Additional file 2: Figure S2).

### Genome-wide transcriptome analysis and functional annotation

Differential expression analysis between the HRFI (inefficient) and LRFI (efficient) groups identified 112 DE annotated genes. The sign of the log_2_(fold change) was used to partition the DE genes into up- and down-regulated groups with 43 DE genes being down-regulated and 69 up-regulated in the LRFI relative to the HRFI groups (Table 2).

**Table 2:** **Genes found to be differentially expressed in the livers of high and low RFI animals**

**Gene ID**	**Locus**	**Mean HRFI, efficient**	**Mean LRFI, inefficient**	**log**_**2**_ (fold change)*	**p-value**	**q-value**
*ABCA3*	25:1796503-1838160	14.089	10.041	−0.489	0.00010	0.0146
*ACE2*	X:135148112-135200083	4.048	7.033	0.797	0.00010	0.0146
*ACTA2*	26:10662362-10679648	43.210	63.910	0.565	0.00010	0.0146
*AGXT2L1*	6:17710759-17730624	12.884	20.459	0.667	0.00005	0.0086
*AKR7A3*	2:133971793-133988579	9.568	14.340	0.584	0.00010	0.0146
*ARHGEF38*	6:20756178-20911532	2.807	1.393	−1.011	0.00005	0.0086
*ATP2A2*	17:56458580-56516270	30.796	21.928	−0.490	0.00040	0.0391
*C1QA*	2:130792854-130795743	332.759	475.615	0.515	0.00015	0.0192
*C1QC*	2:130783985-130788357	288.826	403.344	0.482	0.00035	0.0354
*C28H10orf57*	28:35425156-35433927	69.737	99.972	0.520	0.00005	0.0086
*CA3*	14:79406490-79446489	98.122	150.958	0.622	0.00005	0.0086
*CACNA2D1*	4:38338971-38860701	1.265	0.704	−0.845	0.00010	0.0146
*CFD*	7:45029927-45032847	132.823	179.814	0.437	0.00035	0.0354
*CHPF2*	4:114609573-114641250	4.314	10.102	1.228	0.00005	0.0086
*CHRNE*	19:27117262-27123208	4.823	7.803	0.694	0.00030	0.0311
*CKB*	21:69809411-69812615	15.935	24.904	0.644	0.00005	0.0086
*COL1A1*	19:37088245-37106162	5.848	8.630	0.561	0.00015	0.0192
*COL1A2*	4:11624469-11661163	8.463	11.675	0.464	0.00050	0.0467
*CR2*	16:5253369-5329698	10.291	15.184	0.561	0.00005	0.0086
*CRELD2*	5:120933368-120940337	48.548	34.638	−0.487	0.00030	0.0311
*CST3*	13:42562165-42566091	108.479	149.461	0.462	0.00030	0.0311
*CTGF*	9:70873215-70876451	2.906	4.964	0.772	0.00030	0.0311
*CYBB*	X:111078497-111112510	3.318	5.155	0.636	0.00025	0.0277
*CYP2B6*	18:50564357-50581409	3.226	6.360	0.979	0.00005	0.0086
*CYP4B1*	3:99937027-99957426	4.738	2.662	−0.832	0.00045	0.0434
*CYR61*	3:58678776-58681686	11.769	19.795	0.750	0.00010	0.0146
*EGR1*	7:51438709-51442512	2.865	4.958	0.791	0.00005	0.0086
*EPPK1*	14:2132703-2147067	3.731	2.606	−0.518	0.00010	0.0146
*ERO1LB*	28:8948959-9026767	52.787	33.039	−0.676	0.00005	0.0086
*FABP1*	11:47786225-47793339	1306.810	2103.300	0.687	0.00005	0.0086
*FADS2*	29:41045093-41083225	23.288	43.606	0.905	0.00005	0.0086
*FAM115C*	4:107802455-107821622	1.889	0.854	−1.145	0.00005	0.0086
*FAM174B*	21:14689962-14731037	1.176	3.262	1.472	0.00005	0.0086
*FAM47E*	6:92915864-92950560	2.131	0.979	−1.122	0.00030	0.0311
*FBXL14*	5:108602493-108613689	8.953	6.000	−0.578	0.00005	0.0086
*FCGR3A*	3:7996469-8005232	28.713	43.892	0.612	0.00005	0.0086
*FKBP5*	23:9521253-9643463	22.981	12.855	−0.838	0.00005	0.0086
*FOLR2*	15:52601882-52605587	41.287	56.769	0.459	0.00030	0.0311
*FOS*	10:86883738-86887170	3.668	5.806	0.663	0.00050	0.0467
*GALE*	2:129707102-129711871	29.431	20.571	−0.517	0.00005	0.0086
*GCSH*	18:7793105-7805806	48.733	65.951	0.437	0.00050	0.0467
*GLCE*	10:16009392-16119077	27.221	17.796	−0.613	0.00005	0.0086
*GNG11*	4:11074736-11079756	18.381	26.593	0.533	0.00035	0.0354
*GPC3*	X:17305527-17770816	16.338	8.928	−0.872	0.00005	0.0086
*GPX3*	7:64286947-64295117	30.626	17.682	−0.792	0.00005	0.0086
*GSTM1*	3:33874015-33880642	40.537	61.121	0.592	0.00005	0.0086
*GSTO1*	26:25088447-25097722	16.668	25.758	0.628	0.00005	0.0086
*HDAC10*	5:119814024-119819662	3.337	5.156	0.628	0.00025	0.0277
*HEBP2*	9:77215759-77222555	9.297	13.808	0.571	0.00025	0.0277
*HNF4G*	14:40830784-40965794	18.177	13.052	−0.478	0.00055	0.0499
*HOOK1*	3:86647456-86719014	7.709	4.926	−0.646	0.00005	0.0086
*HOPX*	6:73639365-73649114	8.841	5.224	−0.759	0.00055	0.0499
*HSPB8*	17:58405436-58418688	10.167	15.476	0.606	0.00015	0.0192
*HYOU1*	15:30159426-30171029	41.647	27.360	−0.606	0.00005	0.0086
*IFI27*	21:59330457-59336765	52.798	98.679	0.902	0.00005	0.0086
*IFITM3*	29:51341810-51368123	558.839	816.830	0.548	0.00010	0.0146
*IRF6*	16:75380659-75418348	6.314	9.481	0.586	0.00040	0.0391
*ISG15*	16:52714626-52715654	35.812	59.838	0.741	0.00005	0.0086
*LOC100847320*	27:138114-161660	0.722	1.358	0.912	0.00015	0.0192
*LOC100848726*	29:50712831-50713218	206.496	423.775	1.037	0.00005	0.0086
*LOC100848941*	21:2075268-2167679	23.499	16.438	−0.516	0.00020	0.0239
*LOC510860*	16:4939774-4950979	62.685	88.581	0.499	0.00010	0.0146
*LOC524810*	21:71453611-71596379	9.180	16.524	0.848	0.00005	0.0086
*LOC540627*	26:16130424-16159317	44.807	67.441	0.590	0.00005	0.0086
*LOC786073*	11:107260658-107267533	153.889	225.053	0.548	0.00010	0.0146
*LRRC25*	7:4702830-4708713	6.805	10.810	0.668	0.00010	0.0146
*LST1*	23:27524079-27526993	12.877	21.210	0.720	0.00020	0.0239
*MIR365-2*	19:18810082-18811372	0.825	10.836	3.715	0.00005	0.0086
*MKNK1*	3:100126012-100172590	35.636	25.591	−0.478	0.00015	0.0192
*MSR1*	27:19976239-20058029	10.613	16.751	0.658	0.00005	0.0086
*MYOM1*	24:37673539-37817780	1.312	0.630	−1.058	0.00040	0.0391
*NPC2*	10:86170652-86179237	92.901	132.098	0.508	0.00005	0.0086
*NUFIP1*	12:15163420-15203164	13.200	7.426	−0.830	0.00005	0.0086
*PCDH7*	6:51536696-52011680	2.654	1.318	−1.010	0.00005	0.0086
*PCSK5*	8:52196351-52715122	11.589	8.282	−0.485	0.00010	0.0146
*PGCP*	14:69287216-69893488	2.546	4.513	0.826	0.00015	0.0192
*PRUNE2*	8:52957790-53036819	10.746	14.944	0.476	0.00020	0.0239
*PTGER3*	3:74488088-74589990	7.829	3.859	−1.021	0.00005	0.0086
*PYROXD2*	26:19334004-19359393	13.577	20.835	0.618	0.00020	0.0239
*RGS2*	4:58723599-58724929	5.763	10.749	0.899	0.00005	0.0086
*RN28S1*	3:35428044-35862958	383.558	767.972	1.002	0.00050	0.0467
*RN5-8S1*	25:32467531-32467688	761.308	2520.190	1.727	0.00005	0.0086
*RNASE6*	10:26402505-26404143	13.589	21.187	0.641	0.00005	0.0086
*RNF150*	17:16939480-17220422	4.004	1.299	−1.624	0.00005	0.0086
*ROBO2*	1:24082833-24592295	3.212	2.147	−0.581	0.00020	0.0239
*S100A11*	3:18765878-18770271	37.152	55.245	0.572	0.00015	0.0192
*SALL1*	18:19639132-19657385	12.502	8.288	−0.593	0.00005	0.0086
*SELL*	16:38147609-38173246	9.491	14.272	0.589	0.00005	0.0086
*SFRP2*	17:3829563-3838138	2.129	0.636	−1.743	0.00005	0.0086
*SFTPA1*	28:35850156-35867073	4.006	2.182	−0.877	0.00025	0.0277
*SIGLEC12*	18:57588039-57596060	4.484	7.160	0.675	0.00015	0.0192
*SIX1*	10:73068133-73073934	1.255	0.416	−1.594	0.00005	0.0086
*SLC10A7*	17:11923413-12252718	5.001	3.181	−0.653	0.00015	0.0192
*SLC2A5*	16:45244700-45255826	4.359	0.410	−3.412	0.00005	0.0086
*SLC41A2*	5:68697936-68842834	48.840	31.419	−0.636	0.00005	0.0086
*SLC45A3*	16:3243333-3261978	4.388	2.053	−1.096	0.00005	0.0086
*SLC5A8*	5:65385341-65454064	2.067	0.553	−1.901	0.00005	0.0086
*SMAD1*	17:12877363-12989110	25.185	17.865	−0.495	0.00025	0.0277
*SPTSSB*	1:106851521-106880436	7.439	12.679	0.769	0.00005	0.0086
*TCIRG1*	29:46211734-46223102	20.358	28.700	0.495	0.00045	0.0434
*TGM2*	13:67663047-67697628	12.721	18.074	0.507	0.00015	0.0192
*TM4SF5*	19:27210644-27216978	78.602	107.731	0.455	0.00025	0.0277
*TMSB10*	11:49933203-49934214	647.567	886.612	0.453	0.00055	0.0499
*TMSB4*	1:51022807-51023449	53.115	79.168	0.576	0.00005	0.0086
*TNFRSF8*	16:42437033-42512705	2.533	1.513	−0.743	0.00005	0.0086
*UCP2*	15:54193876-54202724	28.975	41.770	0.528	0.00005	0.0086
*UGGT1*	2:4222092-4333464	10.663	7.574	−0.493	0.00005	0.0086
*UGT2A3*	6:86810491-86838901	12.425	18.879	0.604	0.00005	0.0086
*UGT3A1*	20:38221291-38259719	0.442	1.961	2.151	0.00015	0.0192
*VIM*	13:31944988-31952941	34.456	47.092	0.451	0.00025	0.0277
*WDR35*	11:78912231-78963232	19.525	10.498	−0.895	0.00005	0.0086
*WFDC2*	13:75077193-75084339	15.124	8.167	−0.889	0.00030	0.0311

*Fold change estimates are relative to LRFI, inefficient group.

Six genes that were previously identified in a microarray study that profiled gene expression in the livers of Angus cattle selected for high and low RFI [12] were also identified in this study. The coincident genes included collagen, type I, alpha 1 (*COL1A1*), glutathione S-transferase M1 (*GSTM1*), regulator of G-protein signaling 2 (*RGS2*), ring finger protein 150 (*RNF150*), solute carrier family 2 (facilitated glucose/fructose transporter), member 5 *(SLC2A5)* and vimentin (*VIM*).

Other candidate genes previously described as functioning in the determination of traits related to feed efficiency were also found in this analysis [17-22]. For example, the fatty acid-binding protein 1 (*FABP1*) also known as liver-type fatty acid-binding protein (*L-FABP*) was up-regulated in the LRFI group. Uncoupling protein 2 (mitochondrial, proton carrier) (*UCP2*) and fatty acid desaturase 2 (*FADS2*) with roles in carbohydrate and/or fatty acid metabolism and mitochondrial function were also found to be DE and up-regulated in the LRFI group.

A joint functional annotation analysis using both the up- and down-regulated genes was performed to avoid the potential loss of pathways in which up-regulated genes down-regulate other DE genes and *vice versa*. When analyzed using Database for Annotation, Visualization, and Integrated Discovery (DAVID) v6.7 using cattle as the background [23], the identified functional gene clusters were related to signal, glycoprotein, glycosylation, membrane or transmembrane region, integral to membrane, transport, metal ion binding, regulation of transcription, among others (Additional file 3: Table S1).

**Figure 1:**
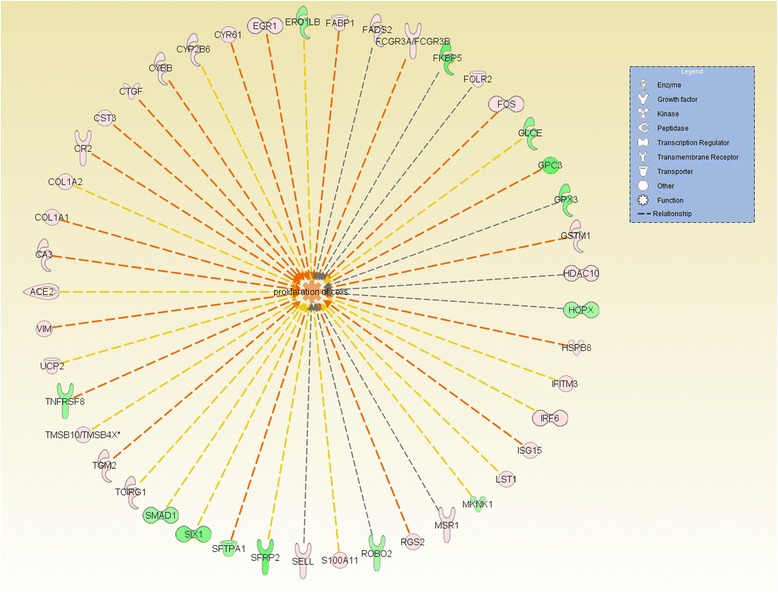
**The differentially expressed gene network with functions in cellular growth and proliferation.** Genes presented in red are up-regulated in the LRFI phenotype group. Genes presented in green are down-regulated in LRFI animals. The intensity of the colors is related to fold change estimates. Arrows presented in orange, gray and yellow indicate activation, effect not predicted and inconsistency, respectively.

The top bio functions identified by QIAGEN’s Ingenuity® Pathway Analysis (IPA®, QIAGEN Redwood City, CA www.qiagen.com/ingenuity) were involved in cellular movement, represented by 28 genes, including *COL1A1*; cytochrome b-245, beta polypeptide (*CYBB*) and *UCP2* and in cell-to-cell signaling and interaction, in which 27 genes were reported as related to this function, including early growth response 1 (*EGR1*), *VIM*, and FBJ murine osteosarcoma viral oncogene homolog (*FOS*). Cellular growth and proliferation (represented by 46 genes including connective tissue growth factor (*CTGF*); *FABP1* and *FADS2 -* Figure 1) and cellular function and maintenance (represented by 23 genes, including surfactant protein A1 (*SFTPA1*) and transglutaminase 2 (*TGM2*)) were also observed. These functions were primarily up-regulated in the LRFI group.

Five KEGG database pathways were found by the DAVID software to be over-represented for genes DE between the divergent RFI groups. These pathways were related to drug or xenobiotic metabolism (BH-adj ≤ 0.44 and BH-adj ≤ 0.27, respectively) complement and coagulation cascades (BH-adj ≤ 0.25) and glutathione (BH-adj ≤ 0.48).

The IPA software reported several other significant canonical pathways involving the 112 DE genes, including complement system (P ≤ 2.16E-05), NRF2-mediated oxidative stress (P ≤ 2.16E-05), melatonin degradation (P ≤ 1.54E-04), glutathione-mediated detoxification (P ≤ 2.08E00), IGF-1 signaling (P ≤ 1.28E-02), TGF-β signaling (P ≤ 7.06E-02), glutathione redox reactions I (P ≤ 8.64E-02) and G-Protein coupled receptor signaling (P ≤ 4.44E-01).

**Figure 2:**
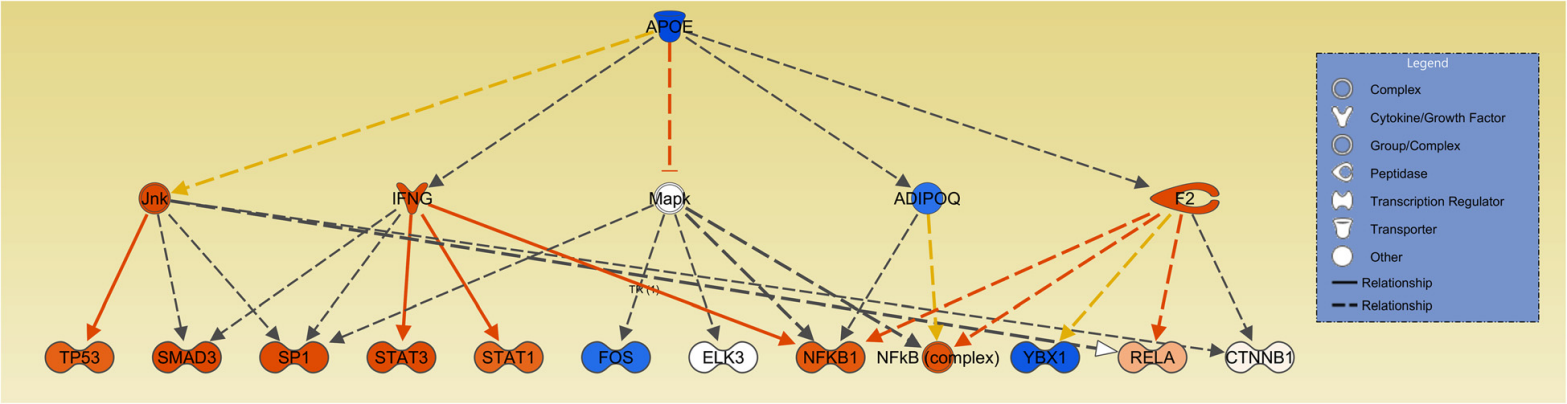
**The mechanistic network of the inferred upstream regulator** ***APOE*** **.** Genes presented in orange are related to genes up-regulated in the LRFI phenotype group. Genes presented in blue are related to genes down-regulated in LRFI animals. The intensity of the colors is related to fold change estimates. Arrows presented in orange, gray and yellow indicate activation, effect not predicted and inconsistency, respectively.

**Figure 3:**
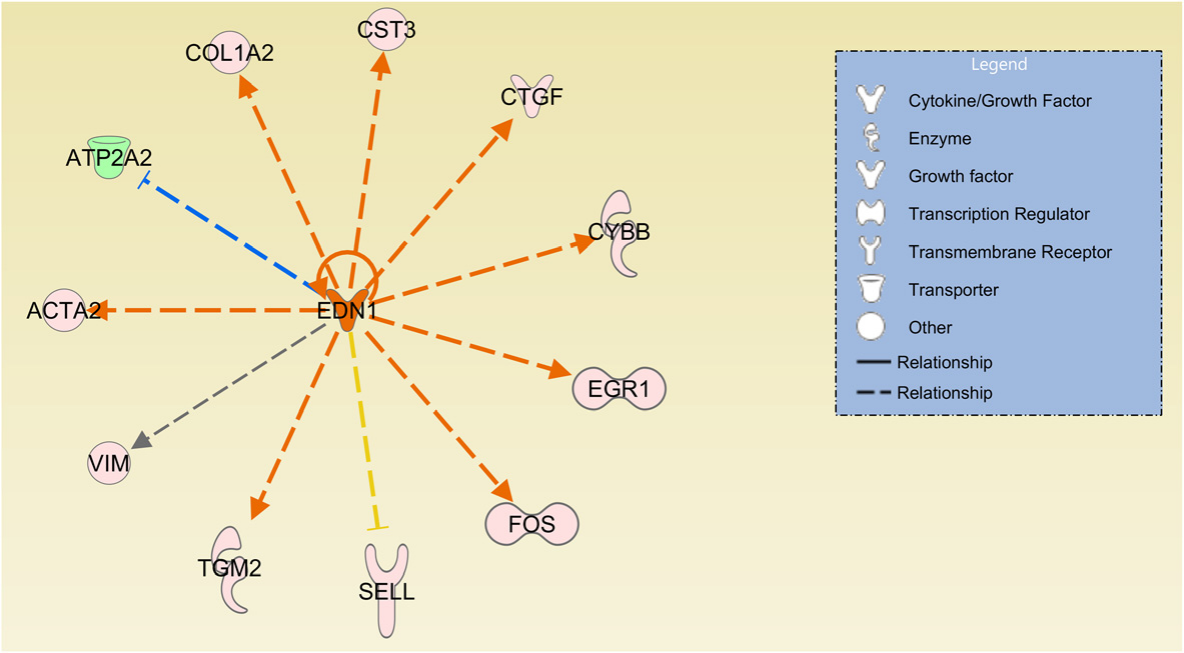
**The differentially expressed gene network of the inferred upstream regulator** ***EDN1*** **.** Genes presented in orange are related to genes up-regulated in the LRFI phenotype group. Genes presented in blue are related to genes down-regulated in LRFI animals. The intensity of the colors is related to fold change estimates. Arrows presented in orange, gray and yellow indicate activation, effect not predicted and inconsistency, respectively.

**Figure 4:**
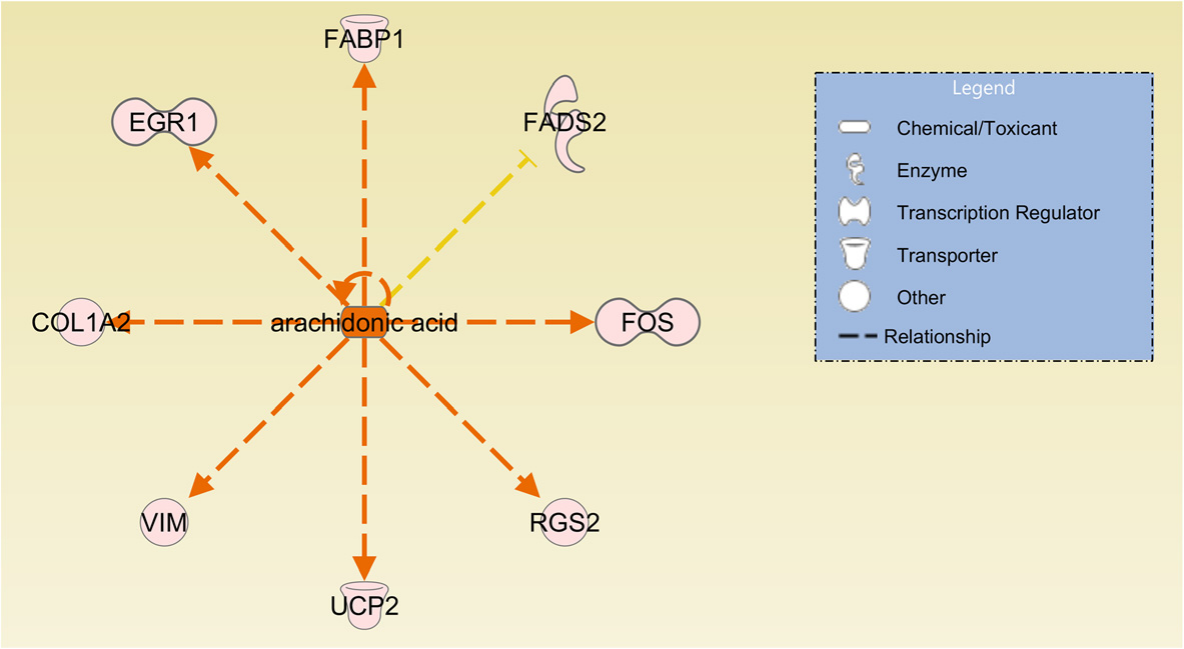
**The differentially expressed gene network of the inferred upstream regulator arachidonic acid.** Genes presented in orange are related to genes up-regulated in the LRFI phenotype group. Genes presented in blue are related to genes down-regulated in LRFI animals. The intensity of the colors is related to fold change estimates. Arrows presented in orange, gray and yellow indicate activation, effect not predicted and inconsistency, respectively.

The upstream regulatory analysis performed by IPA predicted regulators based on the consistency of expression direction changes for DE genes within each pathway (Additional file 4: Table S2). The most important regulators identified in this analysis were apolipoprotein E (*APOE***)** (Figure 2; Additional file 5: Table S3), which was predicted to be inhibited in the LRFI group, endothelin-1 (*EDN1*) (Figure 3; Additional file 6: Table S4) and arachidonic acid (Figure 4; Additional file 7: Table S5) which were predicted to be activated in the LRFI group. Two additional top upstream regulators were inferred: lipopolysaccharide and lysophosphatidic acid, however, it was not possible to infer their activation or inactivation based upon the DE gene set.

The animals comprising the HRFI and LRFI groups were regrouped based on their phenotypes for the component traits dry matter intake (DMI) and average daily gain (ADG). We performed global DE analyses based on these trait groupings (high *vs* low DMI and ADG) to provide insights into the molecular mechanisms that underlie RFI in Nelore beef cattle.

In order to generate differentiated groups for these traits we reduced the sample size to 12 (6 high and 6 low) and 8 (4 high and 4 low) animals for DMI and ADG, respectively, however, we consequently lost some statistical power. Of the 58 DE genes for DMI, 35 were also identified for RFI and of the 39 DE genes for ADG 18 were also DE for RFI. While *ACE2, AGXT2L1, ARHGEF38, CFD, COL1A1, COL1A2, CYP2B6, EGR1, FABP1, FADS2, FAM115C, FKBP5, GLCE, HDAC10, HOOK1, HOPX, IFI27, LOC100848726, LOC100848941, LOC524810, NUFIP1, PCDH7, PTGER3, PYROXD2, RN28S1, RN5-8S1, RNF150, SFRP2, SFTPA1, SIX1, SLC10A7, SLC5A8, SPTSSB* and *WDR35* are likely related to RFI by influencing DMI*, CACNA2D1, CHPF2, CST3, CYR61, FAM115C, FCGR3A, FKBP5, GPC3, HSPB8, IFI27, ISG15, LOC524810, MSR1, RGS2, RNF150, SFRP2, UGT3A1* and *WDR35* influence ADG.

## Discussion

The profitability of beef cattle production is based on both input expenses and output prices for the final products, and these can be used to compute a selection index for feed efficiency [1]. Feed has a major impact on the total cost of beef production systems. It is known that feed efficiency traits are heritable and have sufficient genetic variation within populations to facilitate selection [4-8]. The artificial selection of efficient animals would potentially reduce the cost of cattle production; however, selection for this trait is not easy to implement because it is challenging and expensive to measure individual feed intake on large samples of animals. Residual feed intake, a measure of feed efficiency of growing cattle, is a complex trait controlled by different metabolic processes [9].

The integration of multiple sources of genetic information could potentially explain additional genetic variation via the elucidation of the molecular mechanisms controlling important production traits. Gene expression is a key source of variation between individuals and may be used to identify functional candidate genes and pathways that control target traits. Genes that have previously been identified as being DE in a study of liver tissues of Angus cattle selected for RFI [12] were also found in our analysis. These include *COL1A1*, *GSTM1*, *RGS2*, *RNF150*, *SLC2A5* and *VIM* and suggest that common gene networks underlie RFI regardless of breed genetic background.

Glutathione S-transferase enzymes catalyze the conjugation of glutathione to endogenous compounds such as lipid hydroperoxides and exogenous xenobiotics [24]; the liver is a vital organ for xenobiotic metabolism [25]. The exploration of our genome-wide transcriptome results in DAVID revealed xenobiotic and drug metabolism pathways as being overrepresented and up-regulated in the LRFI group. Chen et al. [12] also found xenobiotic metabolism to be an overrepresented pathway for DE genes, but found this pathway to be down-regulated in the LRFI Angus group in contrast to our findings. Besides *GSTM1* and glutathione S-transferase omega 1 (*GSTO1*) found in our study; other members of the Glutathione S- (GST) family were also reported to be DE by Chen et al. [12]. Genes of the cytochrome P450, family 2, subfamily B, polypeptide 6 (*CYP2B6*) and UDP glucuronosyltransferase 2 family, polypeptide A3 (*UGT2A3*) families were also detected in this pathway. The CYP family and UGTs, which are primarily expressed in liver, encode several enzymes with a crucial function on oxidative metabolism of endogenous substrates, including steroids, fatty acids and exogenous molecules [26,27]. These gene families are also likely involved in the NRF2-mediated oxidative stress response pathway which was consistently found to be up-regulated in the LRFI group by the IPA. While glutathione S-transferase functions in the detoxification of products of oxidative stress, cytochrome P450 proteins catalyze reactions involved in drug metabolism and the synthesis of cholesterol, steroids, and other lipids [26,27]. Our findings suggest that inefficient animals have increased oxidative metabolism possibly stimulated by an increased oxidative stress.

The NRF2-regulated signaling pathway plays a role in protecting mitochondria from oxidative stress during fasting and ensures the efficient utilization of fatty acids in mouse liver. A study has shown that Nrf2-knckout mice are predicted to diminish oxidation and increase the accumulation of lipids in liver due to mitochondrial damage [28]. These findings are also pertinent to broilers, which suggest that genes involved in glutathione metabolism may influence feed efficiency due their function in preserving or improving the activity of certain respiratory chain complexes [29].

Besides NRF2-mediated oxidative stress, IPA also pointed to other pathways overrepresented for DE genes, including, melatonin degradation, IGF-1 signaling, G-Protein coupled receptor signaling, and in agreement with the DAVID results, glutathione redox reactions. The IGF-1 signaling pathway was also found by Chen et al. [12], however, while they found the IGF-Binding Protein 3 (*IGFBP3*) gene to be up-regulated in the LRFI group, we found *CTGF* and *CYR61* genes (cysteine-rich, angiogenic inducer, 61) to be DE in this pathway.

Some of the pathways found in this study, such as IGF-1 signaling have already been reported as functioning in feed efficiency-related traits [30]; however, others are new and may elucidate important unknown mechanisms in Nelore cattle. For example, the involvement of the melatonin degradation pathway in RFI is novel and more studies are necessary to elucidate its role and action in feed efficiency in cattle. Melatonin is responsible for controlling several different biological processes such as a combination of cyclic background and circadian rhythm and also for establishing energy balance and maintaining body weight [31,32]. Its role in energy metabolism and obesity is also recognized [31]; however, the weight-reducing effects of melatonin depend on the actions of several mechanisms, including the circadian clock, energy metabolism and metabolic processes [32]. A functional circadian clock and coordinated metabolic processes are necessary to enhance energy balance and maintenance [32].

The genes of cytochrome P450, families 2 e 4, subfamily B (*CYP4B1*, *CYP2B6*) and UDP glucuronosyltransferase 2 and 3 families, polypeptide A (*UGT3A1* and *UGT2A3*), primarily up-regulated in the LRFI group, were also involved in melatonin degradation. Melatonin putatively attenuates oxidative stress by decreasing lipid peroxidation [33]. Peroxidation of lipids produces aldehyde products which induce the activation of hepatic stellate cells [34]; the primary collagen-producing cells within the liver. Collagen genes were consistently observed as being up-regulated in the LRFI group. Furthermore, melatonin interactions with reactive species are effective against oxidative stress by improving the function of the mitochondrial respiratory chain [35]. Melatonin can increase the levels of several antioxidative enzymes, including glutathione peroxidase and glutathione reductase [33]. Our findings consistently predict the activation of functions important to oxidative processes in the inefficient LRFI group.

The *RGS2* gene was found to be DE between high and low RFI groups in both Nelore and Angus [12] cattle and may affect feed efficiency via its G protein-coupled signaling activity in different cellular functions including the regulation of body weight and adiposity [36]. *RGS2*-knockout mice had lower weight than wild-type controls and exhibited reduced fat deposition, decreased serum lipids and leptin levels, resulting in a lean phenotype even when fed the same diet as control animals, however, food intake and energy expenditure were not altered possibly due to altered energy balance and defects in metabolic processes and energy storage [36]. We found *RGS2* to be up-regulated in the LRFI and in the low ADG groups in agreement with previous reports [12,36]. Furthermore, also supporting our findings, *RGS2* expression has been reported to be up-regulated under conditions of oxidative stress [37].

Many of the enriched functional categories reported by DAVID such as ion transport, metal ion binding, membrane or transmembrane proteins are likely related to the catalysis and transport of substrates through the cell membrane [38]. Transport of substances across cell membranes is required for several vital functions including digestion, absorption of nutrients, cellular signaling, growth, proliferation, cell death and survival which have previously been reported as influencing feed efficiency traits in beef cattle [39]. Some of these biological functions were also found to be enriched for DE genes by the IPA software. Members of the solute carrier group, which are primarily located in the cell membrane (*SLC10A7*, *SLC2A5*, *SLC41A2*, *SLC45A3* and *SLC5A8*), were found to be down-regulated in the HRFI group. The *SLC2A5* gene, which facilitates glucose/fructose transport, was found to be the top up-regulated gene in the HRFI group while genes among the most down-regulated in this group were related to lipid catalysis. These results suggest that efficient animals may have an increased ability to absorb glucose, while inefficient individuals overexpress genes related to the catalysis and intracellular transport of fatty acids. This may indicate that the divergent efficiency groups have preferable sources for obtaining the energy required for maintenance.

Feed intake may influence metabolic activity in liver and consequently energy utilization [18]. Kuhla et al. [18] reported a significant down-regulation of *FABP1* protein in dairy cows that experienced feed restriction and suggested that this may provide a mechanism for limiting fatty acid oxidation and hepatic triacylglyceride accumulation in the event of negative energy balance. These results are supported by a study in which *FABP1* knockout mice demonstrated considerably reduced triacylglyceride levels in liver after fasting [17]. The pattern of *FADS2* gene expression is known to regulate the synthesis of polyunsaturated fatty acids. Moreover, FADS2-deficient mice are resistant to obesity and the dysregulation of lipogenesis [20]. This gene may be also important to the peroxidation susceptibility of lipoproteins and their oxidation rate [40] and was up-regulated in the animals with the highest DMI.

The upstream regulatory analysis performed by IPA, which seeks to identify the upstream transcriptional regulatory cascades that are likely to elucidate the observed changes in gene expression [41], may shed some light on the biological activities that occur in the hepatic tissue of animals that are genetically divergent for RFI. This analysis predicted the top upstream regulators to include *APOE* which was predicted to be inhibited in the LRFI group. The APOE protein functions in lipid transport in liver by assisting in the secretion of very low density lipoprotein (VLDL) [42,43]. Takahashi et al. [43] proposed that serum APOE contents of triglyceride-rich lipoproteins must be controlled by dietary handling in cattle. Wilcox & Heimberg [44] have shown that fasting rats had lower secretions of both VLDL and APOE, therefore having a reduced uptake of VLDL by the liver as compared to fed animals. The inhibition of APOE predicted in the LRFI group may be related to the accumulation of lipoproteins in the liver under conditions of oxidative stress. In a previously performed GWAS study in this population [8], the candidate gene Apolipoprotein A2 (*APOA2*) which functions to stabilize HDL was detected as being associated with RFI.

*EDN1* was also predicted by IPA to be a top upstream regulator of RFI and our results suggest that it is activated in the LRFI group since nine of the eleven DE genes regulated by *EDN1* were found to be coactivated. *EDN1* was inferred by IPA to be a potential regulator of connective tissue growth factor and early growth response genes such as *CTGF* and *EGR1*. Additionally, seven DE genes had expression profiles that were consistent with the activation of arachidonic acid in the LRFI group. These include *FABP1* [45], *UCP2* [46] and *RGS2* [47] which must now be investigated as targets for manipulation through diets containing arachidonic acid. Furthermore, the relative proportion of dietary arachidonic acid to docosahexaenoic acid has been shown to be a determinant of *FADS2* expression and consequently influences polyunsaturated fatty acids metabolism in suckling piglets [48].

Despite the fact that genes found to be DE in this study were not detected in the QTL regions found in a previous GWAS study using the same Nelore population [8]; several common biological mechanisms and key drivers were detected. The majority of QTLs identified in the GWAS lies within gene deserts and may affect feed efficiency via regulatory elements that are yet to be identified involved in the modulation of expression of genes. Non-coding functional elements are poorly understood in cattle and can consist of distal enhancers or transcription factor binding sites. The challenge to interpreting the roles of these QTLs lies in the diversity of function of non-coding variants, poor annotation of regulatory elements and potentially unrecognized control mechanisms [49]. However, candidate genes identified in GWAS are known to cause the DE of genes; when an integrated analysis including both GWAS QTLs and RNA-Seq DE genes was performed using IPA, the differentially expressed transcription factors *EGR1* and *FOS* were suggested to be regulating the candidate gene Plasminogen Activator, Urokinase (*PLAU*) located within a QTL region identified by the GWAS. On the other hand, this gene seems to also coregulate the *VIM* [50] and *CYR61* [51] genes. *EGR1* and *FOS*, found to be up-regulated in the LRFI, are key regulators of genes that are related to cellular growth and differentiation and are also known to be activated in response to oxidative stress [52,53]. Studies targeting the identification of regulatory mutations within the promoters and enhancers/repressors of these genes may be important for understanding the biology of feed efficiency and may have utility for the implementation of genomic selection for feed efficiency in livestock.

Although QTL regions do not have to harbor DE genes, since they can be created by mutations in genes that cause post-translational disruptions affecting the functionality of proteins. The differences in candidate regions/genes found by the GWAS and RNA-Seq may also be explained by the tissue-specific modulation of messenger RNAs (mRNAs). For example, the *HRH4* and *ADAM12* candidate genes located within a QTL region detected by the GWAS could not be tested for expression differences in this study due to their low expression in liver. This finding does not exclude the implication of the DE for these genes in other tissues on feed efficiency.

## Conclusions

We conducted a genome-wide transcriptome profiling study of hepatic tissue from Nelore cattle selected to be genetically divergent for RFI to reveal key metabolic and cell signaling networks. Some previously known mechanisms related to feed efficiency such as xenobiotic metabolism were found; however, new pathways including melatonin degradation were also identified as controlling RFI in Nelore cattle. Overall, our findings demonstrate that changes in gene expression between efficient and inefficient cattle primarily appear to be related to metabolic processes underlying oxidative stress and lipid catabolism. We have potentially identified genes involved in antioxidant mechanisms that play key roles in hepatic metabolic adaptation to oxidative stress. Previous studies have suggested that oxidative stress is increased in inefficient broilers and that this may be related to differences in mitochondrial function [54]. Metabolic response to negative energy balance depends on the availability of fatty acids and ketones as energy sources as well as to the mitochondrial capacity for fatty acid oxidation in tissues with high oxidative energy demands such as liver [55]. The upstream regulators found here guide the future investigation of these molecules to enable the development of intervention strategies such as diet formulation and contribute to the understanding of the physiology and improvement of RFI.

## Methods

### Animals and sampling

All experimental procedures were approved by the Institutional Animal Care and Use Committee Guidelines of the Brazilian Agricultural Research Corporation – EMBRAPA and were sanctioned by the president, Dr. Rui Machado.

These steers comprised half-sib families produced by the artificial insemination of commercial and purebred Nelore dams, derived from 18 sires representing the main breeding lineages commercialized in Brazil. The 83 calves used in this expression study were allocated to feedlots in Embrapa Southeast Research at about 21 months of age. Within the feedlots, animals were maintained either in individual or collective pens and allowed *ad libitum* access to feed and water as described by Oliveira et al. [8]. Briefly, animals were fed twice daily, with diets formulated to contain 40% dry matter (DM) in the form of corn silage; crude protein at 13.5% and energy densities of 2.8. The remaining 60% of DM was concentrate, which comprised ground corn, soybean meal, cotton seed, soybean hulls, limestone, mineral mixture, urea and monensin (Rumensin®). Measures of daily feed intake were collected for at least 70 days and body weight was measured every 14 days.

BLUP estimates of genetic merit for RFI were generated for 585 Nelore steers. Liver samples were available for only 83 of the animals which were ranked according to their additive genetic merit for RFI to select 20 animals that were genetically divergent for RFI, as described below. A relationship matrix computed using pedigree information was used in this analysis. Nelore steers that were genetically divergent for RFI (kg/d) were selected based on BLUP estimates of their additive genetic merits produced using the following model:

$$ y = X\beta + Za + \varepsilon $$

Where, *y* is the vector for average daily feed intake, β is the vector of fixed effects of contemporary group, defined as the combination of season, animal origin and pen type (individual or collective), and partial regressions on age of the animal at entrance to the feedlot, metabolic mid-weight (BW^0.75^) and average daily gain, *a* is the additive genetic merit of the animal for RFI assumed to be normally distributed with E[*a*] = 0 and Var(a) = $\mathrm{A}{\upsigma}_a^2$ where A is the pedigree numerator relationship matrix, and ε is the vector of residual effects inherent to each observation which was assumed to be normally and independently distributed (0, ${\upsigma}_e^2$), X and Z are design matrices for fixed and random effects, respectively. The model was fit by the MIXED procedure of SAS® software; version 9.3 (SAS Institute Inc.) and selected animals were ranked in the most extreme values for additive genetic merit. Where possible, animals that had common sires were sampled from each end of the BLUP distribution.

Dry matter intake and average daily gain described elsewhere [8] were used to decompose RFI via the regrouping of the animals based on these traits for additional gene expression analyses.

### RNA sequencing

Preparation of the mRNA samples for sequencing was performed by ESALQ Genomics Center (Piracicaba, São Paulo, Brazil), using the TruSeq RNA Sample Preparation Kit® (Illumina, San Diego, CA) according to manufacturer’s instructions. Briefly, 100 mg of frozen liver was used to extract RNA using the TRIzol® reagent (Life Technologies, Carlsbad, CA) and 2 μg of total RNA from each liver sample was used for library preparation. The concentration and purity of RNA was measured using NanoDrop™ (Thermos Scientific, Waltham, MA) and then sample integrity was assessed by Bioanalyzer (Agilent, Santa Clara, CA). The mRNA was first enriched from the total RNA by using oligo dT magnetic beads, then the poly(A) RNA was fragmented and cDNA was synthesized. Next, the cDNA underwent end repair, the 3’ ends were adenylated and universal bar-coded adapters were ligated to the cDNA fragments to perform a solid phase PCR to produce the sequencing library. Following library construction, the sequencing library was evaluated and quantified using both an Agilent 2100 Bioanalyzer® and quantitative PCR with the KAPA Library Quantification kit® (KAPA Biosystems, Foster City, CA, USA). Finally, libraries were pooled to perform multiplexing sequencing. Cluster generation and sequencing were performed on the Illumina HiSeq 2000®. Paired-end reads of 2 × 100 bp were produced.

### Processing and alignment of sequence reads

Computations were performed on the HPC resources at the University of Missouri Bioinformatics Consortium (UMBC). Low-quality reads were filtered and adapter sequences trimmed using SeqClean software. TopHat v2.0.6 [13,14] was then used to align the reads to the *Bos taurus* virtual transcriptome internally built by Tophat using the UMD3.1 reference genome. TopHat first extracted the transcript sequences and used Bowtie to align reads to the virtual transcriptome using a provided reference annotation file. The reads that could not be fully mapped to the virtual transcriptome were then mapped to the UMD3.1 reference genome. These reads were converted into genomic mappings and merged with the novel transcriptome mappings and splice junctions in the final output file. A total of 2 mismatches per read were allowed in alignment.

### Transcript assembly and quantification

Cufflinks v2.0.2 [15] was initially used to assemble the aligned reads for each sample individually. Cufflinks assembles the aligned reads and provides a parsimonious set of transcripts as a file. Cufflinks also estimates transcript abundances in Fragments Per Kilobase of exon per Million fragments mapped (FPKM), which normalizes transcript expression for transcript length and the total number of sequence reads per sample. The reference annotation supplied to Cufflinks was used to perform a reference annotation-based transcript assembly. The output for each sample included all reference transcripts as well as novel assembled genes and isoforms. Cufflinks assemblies for all samples were then merged using Cuffmerge v2.0.2 which also runs Cuffcompare internally to classify the transcripts. The available annotation file was provided to this analysis to classify the assembled contigs into novel and known transcripts and to maximize the overall quality of the assembly.

### Testing for differential expression

Cuffdiff2 software was run to test for DE genes between the RFI groups with geometric normalization used to estimate transcript abundance. Correction for multiple testing (q value) was performed using the Benjamini-Hochberg methodology. Cuffdiff2 calculated the FPKM for each transcript, primary transcript, and gene in each sample. A false discovery rate ≤ 0.05 was adopted to consider a gene as being DE.

Data exploration and visualization was performed using the CummeRbund package [14] implemented in the R programming environment.

### Annotation of differentially expressed genes

DAVID v6.7 [23] was used to annotate and interpret the DE gene lists. DAVID software identifies enriched biological themes and gene ontology (GO) terms, clusters functionally related genes and annotation terms for gene lists with EASE scores < 0.1. The Functional Annotation Tool was used to determine the most relevant GO terms within each list of DE genes. The Functional Annotation Clustering algorithm was used to generate a report of related annotation terms and groups of annotation clusters. Finally, DAVID Pathway was used to map the enriched pathways in which DE genes are involved, using the KEGG database.

The IPA (www.qiagen.com/ingenuity) was also used to discover and explore biological processes and the roles of DE genes. The Ingenuity Pathways Knowledge Base comprises relationships such as between genes, mRNAs and proteins to test for significantly overrepresented networks and pathways. We provided the fold changes and q-values of DE among genes from the Cuffdiff analysis to the IPA to perform the statistical analysis for the representation of each network and to visualize the results.

## Availability of supporting data

The RNA-seq data sets supporting the results of this study are available in the ENA repository (EMBL-EBI), under accession PRJEB7696.

## Additional files

Additional file 1: Figure S1. Boxplot of the log_10_ of FPKM (Fragments Per Kilobase of exon per Million fragments mapped) expression values for both RFI groups. Additional file 2: Figure S2. Principal Component Analysis (PCA) between the RFI treatments for all gene-level features. Additional file 3: Table S1. Enriched GO terms from DAVID software for differentially expressed genes. Additional file 4: Table S2. Upstream regulators identified by QIAGEN’s Ingenuity® Pathway Analysis. Additional file 5: Table S3. Mechanistic network of the inferred upstream regulator *APOE*. Additional file 6: Table S4. Mechanistic network of the inferred upstream regulator *EDN1*. Additional file 7: Table S5. Mechanistic network of the inferred upstream regulator arachidonic acid.
