# Supplementary figures and images for: Global liver gene expression differences in Nelore steers with divergent residual feed intake phenotypes
Source: BMC Genomics. 2015 Mar 25;16(1):242. doi: 10.1186/s12864-015-1464-x (PMC4381482; doi:10.1186/s12864-015-1464-x)

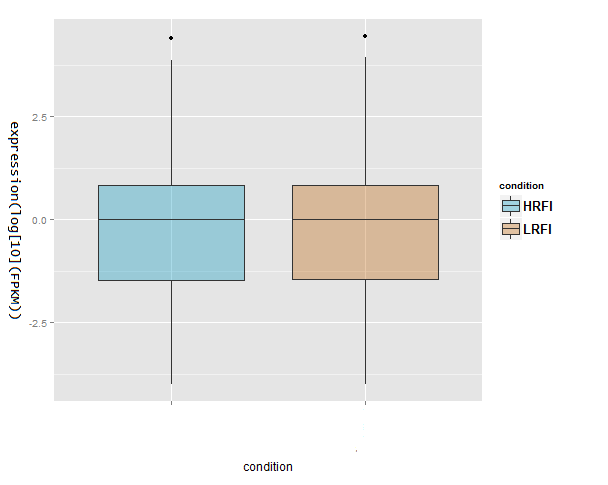

Supplement: Additional file 1: Figure S1. — Boxplot of the log10 of FPKM (Fragments Per Kilobase of exon per Million fragments mapped) expression values for both RFI groups. [file 12864_2015_1464_MOESM1_ESM.tiff]

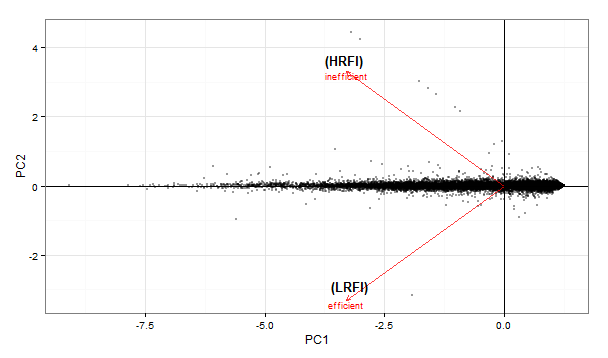

Supplement: Additional file 2: Figure S2. — Principal Component Analysis (PCA) between the RFI treatments for all gene-level features. [file 12864_2015_1464_MOESM2_ESM.tiff]
